# Using Artificial Intelligence as a Diagnostic Decision Support Tool in Skin Disease: Protocol for an Observational Prospective Cohort Study

**DOI:** 10.2196/37531

**Published:** 2022-08-31

**Authors:** Anna Escalé-Besa, Aïna Fuster-Casanovas, Alexander Börve, Oriol Yélamos, Xavier Fustà-Novell, Mireia Esquius Rafat, Francesc X Marin-Gomez, Josep Vidal-Alaball

**Affiliations:** 1 Centre d'Atenció Primària Navàs-Balsareny Institut Català de la Salut Navàs Spain; 2 Unitat de Suport a la Recerca de la Catalunya Central Fundació Institut Universitari per a la Recerca a l'Atenció Primària de Salut Jordi Gol i Gurina Sant Fruitós de Bages Spain; 3 Health Promotion in Rural Areas Research Group Gerència Territorial de la Catalunya Central Institut Català de la Salut Sant Fruitós de Bages Spain; 4 iDoc24 Inc San Francisco, CA United States; 5 Institute of Clinical Sciences Sahlgrenska Academy University of Gothenburg Gothenburg Sweden; 6 Dermatology Department Hospital de la Santa Creu i Sant Pau Universitat Autònoma de Barcelona Barcelona Spain; 7 Fundació Althaia de Manresa Manresa Spain; 8 Servei d’Atenció Primària Osona Gerència Territorial de Barcelona Institut Català de la Salut Vic Spain; 9 Faculty of Medicine University of Vic-Central University of Catalonia Vic Spain

**Keywords:** machine learning, artificial intelligence, data accuracy, computer-assisted diagnosis, neural network computer, support tool, skin disease, cohort study, dermatology

## Abstract

**Background:**

Dermatological conditions are a relevant health problem. Each person has an average of 1.6 skin diseases per year, and consultations for skin pathology represent 20% of the total annual visits to primary care and around 35% are referred to a dermatology specialist. Machine learning (ML) models can be a good tool to help primary care professionals, as it can analyze and optimize complex sets of data. In addition, ML models are increasingly being applied to dermatology as a diagnostic decision support tool using image analysis, especially for skin cancer detection and classification.

**Objective:**

This study aims to perform a prospective validation of an image analysis ML model as a diagnostic decision support tool for the diagnosis of dermatological conditions.

**Methods:**

In this prospective study, 100 consecutive patients who visit a participant general practitioner (GP) with a skin problem in central Catalonia were recruited. Data collection was planned to last 7 months. Anonymized pictures of skin diseases were taken and introduced to the ML model interface (capable of screening for 44 different skin diseases), which returned the top 5 diagnoses by probability. The same image was also sent as a teledermatology consultation following the current stablished workflow. The GP, ML model, and dermatologist’s assessments will be compared to calculate the precision, sensitivity, specificity, and accuracy of the ML model. The results will be represented globally and individually for each skin disease class using a confusion matrix and one-versus-all methodology. The time taken to make the diagnosis will also be taken into consideration.

**Results:**

Patient recruitment began in June 2021 and lasted for 5 months. Currently, all patients have been recruited and the images have been shown to the GPs and dermatologists. The analysis of the results has already started.

**Conclusions:**

This study will provide information about ML models’ effectiveness and limitations. External testing is essential for regulating these diagnostic systems to deploy ML models in a primary care practice setting.

## Introduction

Health care systems in Western countries are increasingly exposed to new challenges: a high volume of demand, aging populations, chronic diseases, a high degree of comorbidity, and the global pandemic situation. These factors, together with the lack of professionals, particularly general practitioners (GPs), generate the need to find new solutions to improve the quality of care and the workflow of professionals [1].

Dermatological conditions are a relevant health problem, and skin disease is one of the principal reasons why patients visit their GPs. Every person has on average 1.6 skin diseases per year [2]. About 20% of all GP visits are related to a dermatological concern, and 15% of all telehealth visits are related to dermatology [3,4]. About 7.6% of the total population of Catalonia visit a primary care center (PCC) due to skin concerns every year, and around 35% are referred to a dermatology specialist [5,6]. Nowadays, in the health care area of central Catalonia, teledermatology consultations are commonly used to refer patients to a hospital-based dermatologist. It is estimated that more than 70% of all PCC patients with a skin problem can be effectively triaged with teledermatology and do not need a face-to-face visit with a dermatologist [7,8].

The use of computer-assisted diagnosis in medicine dates to the 1960s in radiology. The initial description of artificial intelligence (AI) in dermatopathology dates to 1987, when the text-based system TEGUMENT was produced [9]. TEGUMENT included a semantic tree with 986 potential diagnoses used to assist the dermatologist in the histopathologic differential diagnosis of diseases and tumors of the skin. Computer-aided melanoma diagnosis was introduced in the early 2000s in dermatology using rule-based classifiers, which use predefined features to classify images into desired categories [10].

The application of teledermatology worldwide has increased over the years. It is used in many PCC settings and has been well established and backed by extensive research that it is a viable method of triage, particularly for skin cancer lesions [11]. Studies comparing the general accuracy of face-to-face dermatology consultation versus teledermatology have different results. In general, face-to-face consultations achieve higher diagnostic accuracy than teledermatology. However, some studies did report the high accuracy of teledermatology diagnoses for skin cancer [12]. Nevertheless, it is necessary to first ensure that the clinicians have high interrater reliability; without this, it is difficult to tell whether the limited agreement in diagnoses is related to the use of the technology itself or differences in clinical opinion that ordinarily exist in practice. In this context, studies have compared the diagnostic agreement between GPs using telemedicine and dermatologists. The results of the studies showed an overall diagnostic agreement of 65.52%, showing that GPs tend to overdiagnose some diseases [13]. The concordance obtained for teledermatology was 94.7%. Even though this technique showed merits in triage quality, it presented low accuracy in inflammatory problems [13]. Teledermatology has the potential to increase access by facilitating referrals and offering convenience and decreased waiting times, as well as providing diagnostic support and improved satisfaction for both patients and providers [8,14-17]. To achieve the correct implementation of AI in primary care, it is important to know the real needs and developed an easy-to-use interface, which can help reduce resistance to change from traditional to touch-based interfaces in current clinical setups [18].

In recent years, AI has been developed, researched, and applied in many medical disciplines. Images are the most commonly used form of data for AI development, such as electrocardiograms or radiologic images [19-21]. Dermatopathology is particularly suited for deep learning algorithms, because pattern recognition in scanning magnification is fundamental for diagnosis [10,22-24]. Furthermore, machine learning (ML) is increasingly being applied to dermatology, particularly focused on skin cancer detection using image analysis with ML models that include deep convolutional neural networks (CNNs) [25,26]. Algorithms and models that include CNNs were introduced in the 1980s [23], but it was not until 2012 that the ImageNet competition demonstrated their potential for image analysis. Since then, CNN has become a popular ML approach in several disciplines including dermatology [27]. There are also ML studies that have investigated the use of a wider classification of skin diseases that could be used in primary health care [28]. The evolution in ML came around 2010 with deep learning [10], and it has revolutionized tasks such as image classification and segmentation and speech recognition.

Even though GPs see a lot of skin ailments [5,29], few studies have been conducted in primary health care settings prospectively. However, some studies have included GPs along with dermatologists as readers for the comparison group to compare the performance of ML with clinicians [11,28,30] and have concluded that AI tools could be used in primary care [28]. For all these reasons, the main objective of the study is to perform a prospective validation in real primary care practice settings of an ML model as a diagnostic decision support tool for the diagnosis of dermatological conditions in a rural area of Catalonia (Spain).

## Methods

### Study Design

#### Trial Design

This is a prospective study that aims to evaluate an ML model’s performance, comparing its diagnostic capacity with GPs and dermatologists. A secure, anonymous, and stand-alone web interface that is compatible to any mobile device was integrated with the Autoderm application programming interface (API; iDoc24 Inc).

To conduct this study, the following procedure were carried out until the required number of samples was reached: (1) a suitable patient with skin concern was asked to participate and sign the patient study agreement; (2) GPs diagnosed the skin condition; (3) GPs took 1 good-quality image of the skin condition; (4) GPs sent the photograph as a teledermatology consultation following the current workflow; (5) the image were entered into the Autoderm ML interface; and (6) dermatologists diagnosed the skin condition.

The satisfaction of the health care professionals using the ML tool were assessed using 3 questions embedded in the questionnaire. The questions relate to the potential usefulness of the tool to help the diagnosis or consider further diagnosis not contemplated initially and the potential use of the tool to avoid a dermatology referral.

#### Study Population, Site Participation, and Recruitment

The study was conducted in PCCs managed by the Catalan Health Institute (the main primary care services provider in Catalonia) in central Catalonia, which includes the regions of Anoia, Bages, Moianès, Berguedà, and Osona. The reference population included in the study was around 512,050 habitants. The recruitment of prospective subjects was done consecutively.

### Data Collection and Sources of Information

#### Patients, Data Collection, Sources of Information, and Intervention

GPs collected data from consecutive patients who met the inclusion criteria after obtaining written informed consent. The collected data were reported exclusively in a case report form.

The GP diagnosed the skin condition and filled in a questionnaire. For each patient, the GP used a smartphone camera to take a close-up good-quality image of the skin problem. The image is anonymous, as it is not possible to identify the patients. The GP then used the Autoderm ML interface to upload the anonymized image and filled in the questionnaire with the top 5 diagnoses generated by the ML model.

This evaluation study of the Autoderm API tool is intended as a validation study of a tool to support the diagnosis of skin lesions in real clinical practice conditions in primary care. Therefore, although the tool uses a closed source code, this study is intended to be a starting point to see if similar tools can be suitable for use as working tools in real clinical conditions. Autoderm is a research-backed, Conformité Européenne–marked dermatology search engine using ML technology to help provide faster and more accurate skin diagnosis. The current ML model can screen for 44 different skin disease types, which includes inflammatory skin diseases, skin tumors, and genital skin concerns, and can be accessed via an API. For this study, a user web interface was developed for the easy upload of images from the smartphone library or those taken with the smartphone camera. From just a smartphone photo, the model generates the top 5 ranked skin diseases in order of probability. The life cycle of this ML model is estimated to be around 3 months. After this period, the model will be upgraded to a more accurate model that will possibly include more skin diseases.

At its current stage, the ML model uses a 34-layer pretrained ResNet model provided by TorchVision (PyTorch) that is used for applications such as computer vision and natural language processing. In addition, the model has been trained using transfer learning on a proprietary data set of 55,364 images for the training set and 13,841 images for testing. The average accuracy of the model used is 31.7% for the top 1 diagnosis and 68.1% for the top 5. Some skin diseases have higher accuracy and some have lower accuracy, which is a consequence of the number of images the ML was trained on and the fact that some skin diseases are more distinct and certain anatomic locations make diagnosis more difficult. Before deployment, the ML model was also manually tested with a data set collected from various websites that provided images of skin disease taken with a mobile camera. The ML model was deployed when it was deemed to be robust. The 44 different skin disease classes represent about 90% of what the general public are concerned about and consults for.

To get a second opinion, the GP incorporated the anonymized image and an accurate description of the skin lesion into the patient’s medical history following the current teledermatology workflow. The dermatologist then filled in the “Assessment by teledermatology” questionnaire after receiving the information. The response time was expected to be about 2-7 days.

In case of a dermatology referral, the GP filled in the “Assessment by in-person dermatologist” questionnaire by accessing the electronic health records as they become available. The average waiting time for a dermatology referral ranges from 30-90 days.

The questionnaire case number was predefined before the initiation of the data collection phase and was the same for all questionnaires, making it impossible to identify the patient.

#### Inclusion Criteria

Patients visiting for reasons related to a cutaneous disease at a participating PCC, who provided written informed consent and were aged ≥18 years, were included in the prospective study.

#### Exclusion Criteria

Patients with a cutaneous lesion that could not be photographed with a smartphone or had conditions associated with a risk of poor protocol compliance were excluded from the study. Images with poor quality were also excluded from the study.

### Statistical Analysis

#### Calculation of Sample Size

To compare the performance of the ML model with those of the GPs and dermatologists, a sample size of 100 images of skin diseases from patients who meet the inclusion criteria is required. The proposed sample size is based on sample size calculation used in similar research studies [31-33].

#### Planned Analysis

The validation data set will include about 100 cases, consisting of an image and 3 or 4 assessments: the face-to-face assessment by a GP, the assessment made by teledermatology, the top 5 differential diagnoses from the ML model ordered by probability, and the assessment by the face-to-face dermatologist (in cases with a referral). The ML model assessment will be limited to 44 skin diseases classes. A confusion matrix will be used to calculate the precision, sensitivity (recall), specificity, and accuracy of the ML model. For each individual skin disease, the number of true positives, true negatives, false positives, and false negatives will be calculated. To evaluate the ML multiclass classifier, data will be treated as a collection of binary problems, 1 for each skin disease class. Area under the curve and receiver operating characteristics curve for N number of skin diseases classes will be calculated using one-versus-all methodology. Macro- and micro-averaging measures will be considered to highlight the performance of infrequent skin disease classes (weighted by prevalence). Precision, recall, and *F*-measure will be calculated independently for each skin disease class, and the results will be combined to obtain the average precision and *F*-score. The accuracy of the top 3 diagnoses of the ML model will be also calculated.

### Ethics Approval

The Institut Universitari d'Investigació en Atenció Primària (University Institute for Research in Primary Health Care) Jordi Gol i Gurina ethics committee approved the trial study protocol (code 20-159P). Written informed consent was sought from all patients participating in the study.

## Results

The results will be represented globally and individually for each skin disease class using a confusion matrix and one-versus-all methodology. The time taken to make the diagnosis will also be taken into consideration. The satisfaction of the professionals with the use of this ML tool will be assessed.

Patient recruitment began in June 2021 and lasted for 5 months. Currently, all patients have been recruited and the images have been shown to the GPs and dermatologists. The analysis of the results has already started. We hope that sufficient evidence can be obtained to validate this image analysis ML model. We believe the results will be used in clinical practice on patients with skin diseases to make a GP’s workflow more efficient and safer for the patient. This study is a first approach to designing larger ML model validation studies.

It has to be considered that even if the ML model does not provide a better diagnosis than the doctor’s, it is expected to help the practitioner consider other differential diagnoses.

## Discussion

This study aims to perform a prospective validation of an ML model as a diagnostic decision support tool for the diagnosis of dermatological conditions. It would also assess the diagnostic accuracy and efficacy of a ML model in a PCC setting. In this context, this study may provide added value for both patients and primary care physicians, increasing the effectiveness and efficiency of the system, and will provide information about ML models’ effectiveness and limitations. External testing is essential to regulate these diagnostic systems and deploy ML models in real PCC settings.

First, the most relevant limitation of this study is the number of image samples used for the evaluation of the performance of the ML model. As Autoderm assesses only 44 skin diseases and that the prevalence of a substantial number of these skin conditions represents less than 1% to 5% of the images, the sample data of each class may be unbalanced and some skin conditions may not be evaluated, causing an insufficient confidence level and therefore, less conclusive results for these specific conditions.

Second, due to the sample size and consecutive case recruitment, we will probably not obtain representative results for less common diseases. As class imbalance may be an issue in the 100 patients recruited, we will focus on the *F*-Score for the analysis, as otherwise having 90% of the most common skin lesions may overestimate the quality of the model when considering accuracy, sensitivity, and specificity. It has to be taken into consideration that this study will be done in real practice conditions, and we will not be able to select the patients.

Third, a diagnosis made with only 1 image with the most optimal composition may present inherent limitations compared to diagnoses made in a clinical setting. Our ML algorithm output was based on a single photograph, which differs from other ML algorithms that consider more than 1 photograph and even those with the same algorithm available for the general public that considers 2 images.

Fourth, another limitation is that our data will not include additional testing and only a subset of suspected malignancies will have a biopsy confirmation. Instead, our golden standard for each case is based on aggregating the differential diagnoses of a panel of dermatologists. Ambiguities in diagnosis do exist in clinical practice, which makes it challenging to evaluate the accuracy of clinicians and deep learning systems, especially for conditions such as rashes, which are not typically biopsied.

Fifth, our ML algorithm did not include additional clinical metadata (past medical history, symptoms, appearance, and the texture), which is a probable grievance when comparing ML versus physicians’ diagnostic accuracy.

Lastly, the clinicians were requested to provide just the top 3 diagnosis, even if they had other potential options.

## Abbreviations

AI: artificial intelligence
API: application programming interface
CNN: convolutional neural network
GP: general practitioner
PCC: primary care center

## Data Availability

Our manuscript is based on confidential and sensitive health data. However, to support scientific transparency, we will publish deidentified data for reviewers or for replication purposes. The data will be deposited and made available in our publicly accessible Mendeley repository.

## References

[1] Sánchez-Sagrado T (2013). Are there too many or too few physicians in Spain? migration: the eternal resource. Rev Clin Esp (English Ed).

[2] Lim HW, Collins SAB, Resneck JS, Bolognia JL, Hodge JA, Rohrer TA, van Beek MJ, Margolis DJ, Sober AJ, Weinstock MA, Nerenz DR, Smith Begolka W, Moyano JV (2017). The burden of skin disease in the United States. J Am Acad Dermatol.

[3] Schofield J K, Fleming D, Grindlay D, Williams H (2011). Skin conditions are the commonest new reason people present to general practitioners in England and Wales. Br J Dermatol.

[4] Tensen E, van der Heijden J P, Jaspers M W M, Witkamp L (2016). Two decades of teledermatology: current status and integration in national healthcare systems. Curr Dermatol Rep.

[5] Servei Català de la Salut (2013). Activitat assistencial de la xarxa sanitària de Catalunya, any 2012: registre del conjunt mínim bàsic de dades (CMBD). Barcelona: Departament de Salut.

[6] Lowell BA, Froelich CW, Federman DG, Kirsner RS (2001). Dermatology in primary care: prevalence and patient disposition. J Am Acad Dermatol.

[7] Porta N, San Juan J, Grasa M, Simal E, Ara M, Querol I (2008). Diagnostic agreement between primary care physicians and dermatologists in the health area of a referral hospital. Actas Dermosifiliogr (English Ed).

[8] López Seguí Francesc, Franch Parella J, Gironès García Xavier, Mendioroz Peña Jacobo, García Cuyàs Francesc, Adroher Mas C, García-Altés Anna, Vidal-Alaball J (2020). A cost-minimization analysis of a medical record-based, store and forward and provider-to-provider telemedicine compared to usual care in Catalonia: more agile and efficient, especially for users. Int J Environ Res Public Health.

[9] Potter B, Ronan SG (1987). Computerized dermatopathologic diagnosis. J Am Acad Dermatol.

[10] Talebi-Liasi Faezeh, Markowitz O (2020). Is artificial intelligence going to replace dermatologists?. Cutis.

[11] Börve Alexander, Dahlén Gyllencreutz Johan, Terstappen K, Johansson Backman Eva, Aldenbratt A, Danielsson M, Gillstedt M, Sandberg C, Paoli J (2015). Smartphone teledermoscopy referrals: a novel process for improved triage of skin cancer patients. Acta Derm Venereol.

[12] Finnane A, Dallest K, Janda M, Soyer HP (2017). Teledermatology for the diagnosis and management of skin cancer: a systematic review. JAMA Dermatol.

[13] Ferrer Rosa Taberner, Bezares Antonio Pareja, Mañes Alex Llambrich, Mas Antonia Vila, Gutiérrez Ignacio Torné, Lladó Cristina Nadal, Estaràs Guillermo Mas (2009). Diagnostic reliability of an asynchronous teledermatology consultation. Article in Spanish. Aten Primaria.

[14] Mounessa JS, Chapman S, Braunberger T, Qin R, Lipoff JB, Dellavalle RP, Dunnick CA (2018). A systematic review of satisfaction with teledermatology. J Telemed Telecare.

[15] Vidal-Alaball J, Álamo-Junquera Dolores, López-Aguilá Sílvia, García-Altés Anna (2015). Evaluation of the impact of teledermatology in decreasing the waiting list in the Bages region (2009-2012). Article in Spanish. Aten Primaria.

[16] Vidal-Alaball J, López Seguí Francesc, Garcia Domingo JL, Flores Mateo G, Sauch Valmaña Gloria, Ruiz-Comellas A, Marín-Gomez Francesc X, García Cuyàs Francesc (2020). Primary care professionals' acceptance of medical record-based, store and forward provider-to-provider telemedicine in Catalonia: results of a web-based survey. Int J Environ Res Public Health.

[17] Lee MK, Rich K (2021). Who is included in human perceptions of AI?: trust and perceived fairness around healthcare AI and cultural mistrust.

[18] Calisto FM, Ferreira A, Nascimento JC, Gonçalves D (2017). Towards touch-based medical image diagnosis annotation.

[19] Calisto FM, Santiago C, Nunes N, Nascimento JC (2021). Introduction of human-centric AI assistant to aid radiologists for multimodal breast image classification. Int J Hum Comput Stud.

[20] Calisto FM, Nunes N, Nascimento JC (2020). BreastScreening: on the use of multi-modality in medical imaging diagnosis.

[21] Attia Zachi I, Harmon David M, Behr Elijah R, Friedman Paul A (2021). Application of artificial intelligence to the electrocardiogram. Eur Heart J.

[22] Wells A, Patel S, Lee JB, Motaparthi K (2021). Artificial intelligence in dermatopathology: diagnosis, education, and research. J Cutan Pathol.

[23] Thomsen K, Iversen L, Titlestad TL, Winther O (2020). Systematic review of machine learning for diagnosis and prognosis in dermatology. J Dermatolog Treat.

[24] Du AX, Emam S, Gniadecki R (2020). Review of machine learning in predicting dermatological outcomes. Front Med (Lausanne).

[25] Gomolin A, Netchiporouk E, Gniadecki R, Litvinov IV (2020). Artificial intelligence applications in dermatology: where do we stand?. Front Med (Lausanne).

[26] Esteva A, Kuprel B, Novoa RA, Ko J, Swetter SM, Blau HM, Thrun S (2017). Dermatologist-level classification of skin cancer with deep neural networks. Nature.

[27] Morid MA, Borjali A, Del Fiol G (2021). A scoping review of transfer learning research on medical image analysis using ImageNet. Comput Biol Med.

[28] Liu Y, Jain A, Eng C, Way DH, Lee K, Bui P, Kanada K, de Oliveira Marinho G, Gallegos J, Gabriele S, Gupta V, Singh N, Natarajan V, Hofmann-Wellenhof R, Corrado GS, Peng LH, Webster DR, Ai D, Huang SJ, Liu Y, Dunn RC, Coz D (2020). A deep learning system for differential diagnosis of skin diseases. Nat Med.

[29] Servei Català de la Salut Activitat assistencial de la xarxa sanitària de Catalunya, any 2012. Departament de Salut.

[30] Tschandl P, Codella N, Akay BN, Argenziano G, Braun RP, Cabo H, Gutman D, Halpern A, Helba B, Hofmann-Wellenhof R, Lallas A, Lapins J, Longo C, Malvehy J, Marchetti MA, Marghoob A, Menzies S, Oakley A, Paoli J, Puig S, Rinner C, Rosendahl C, Scope A, Sinz C, Soyer HP, Thomas L, Zalaudek I, Kittler H (2019). Comparison of the accuracy of human readers versus machine-learning algorithms for pigmented skin lesion classification: an open, web-based, international, diagnostic study. Lancet Oncol.

[31] Kamulegeya LH, Okello M, Bwanika JM, Musinguzi D, Lubega W, Rusoke D, Nassiwa F, Börve A Using artificial intelligence on dermatology conditions in Uganda: a case for diversity in training data sets for machine learning. bioRxiv..

[32] Brinker TJ, Hekler A, Enk AH, Berking C, Haferkamp S, Hauschild A, Weichenthal M, Klode J, Schadendorf D, Holland-Letz T, von Kalle C, Fröhling Stefan, Schilling B, Utikal JS (2019). Deep neural networks are superior to dermatologists in melanoma image classification. Eur J Cancer.

[33] Haenssle HA, Fink C, Schneiderbauer R, Toberer F, Buhl T, Blum A, Kalloo A, Hassen ABH, Thomas L, Enk A, Uhlmann L, Alt Christina, Arenbergerova Monika, Bakos Renato, Baltzer Anne, Bertlich Ines, Blum Andreas, Bokor-Billmann Therezia, Bowling Jonathan, Braghiroli Naira, Braun Ralph, Buder-Bakhaya Kristina, Buhl Timo, Cabo Horacio, Cabrijan Leo, Cevic Naciye, Classen Anna, Deltgen David, Fink Christine, Georgieva Ivelina, Hakim-Meibodi Lara-Elena, Hanner Susanne, Hartmann Franziska, Hartmann Julia, Haus Georg, Hoxha Elti, Karls Raimonds, Koga Hiroshi, Kreusch Jürgen, Lallas Aimilios, Majenka Pawel, Marghoob Ash, Massone Cesare, Mekokishvili Lali, Mestel Dominik, Meyer Volker, Neuberger Anna, Nielsen Kari, Oliviero Margaret, Pampena Riccardo, Paoli John, Pawlik Erika, Rao Barbar, Rendon Adriana, Russo Teresa, Sadek Ahmed, Samhaber Kinga, Schneiderbauer Roland, Schweizer Anissa, Toberer Ferdinand, Trennheuser Lukas, Vlahova Lyobomira, Wald Alexander, Winkler Julia, Wölbing Priscila, Zalaudek Iris, Reader study level-I and level-II Groups (2018). Man against machine: diagnostic performance of a deep learning convolutional neural network for dermoscopic melanoma recognition in comparison to 58 dermatologists. Ann Oncol.

